# Using self-determination theory to understand and improve recruitment for the Coaching for Healthy Ageing (CHAnGE) trial

**DOI:** 10.1371/journal.pone.0259873

**Published:** 2021-11-19

**Authors:** Abby Haynes, Catherine Sherrington, Geraldine Wallbank, James Wickham, Allison Tong, Catherine Kirkham, Shona Manning, Elisabeth Ramsay, Anne Tiedemann

**Affiliations:** 1 Institute for Musculoskeletal Health, The University of Sydney and Sydney Local Health District, Sydney, NSW, Australia; 2 School of Public Health, Faculty of Medicine and Health, The University of Sydney, Sydney, NSW, Australia; 3 School of Biomedical Sciences, Charles Sturt University, Orange, NSW, Australia; 4 Centre for Kidney Research, The Children’s Hospital at Westmead, Westmead, NSW, Australia; 5 Christian Homes Tasmania Inc, Kingston, TAS, Australia; Federation University Australia, AUSTRALIA

## Abstract

**Background:**

Intervention trials promoting physical activity among older people frequently report low and unrepresentative recruitment. Better understanding of reasons for participation can help improve recruitment. This study explored why participants enrolled in the Coaching for Healthy Ageing (CHAnGE) trial, including how their decision was influenced by recruitment strategies. CHAnGE was a cluster randomised controlled trial testing the effectiveness of a healthy ageing program targeting inactivity and falls. Seventy-two groups of people aged 60+ were recruited from community organisations via informal presentations by the health coaches.

**Methods:**

We conducted a secondary thematic analysis of interview data from our wider qualitative evaluation in which 32 purposively sampled trial participants took part in semi-structured interviews about their experiences of CHAnGE. Data relating to recruitment and participation were analysed inductively to identify themes, then a coding framework comprising the core constructs from self-determination theory—autonomy, competence and relatedness—was used to explore if and how this theory fit with and helped to explain our data.

**Results:**

Recruitment presentations promoted the CHAnGE intervention well in terms of addressing value expectations of structured support, different forms of accountability, credibility, achievability and, for some, a potential to enhance social relationships. Participation was motivated by the desire for improved health and decelerated ageing, altruism and curiosity. These factors related strongly to self-determination concepts of autonomy, competence and relatedness, but the intervention’s demonstrated potential to support self-determination needs could be conveyed more effectively.

**Conclusions:**

Findings suggest that recruitment could have greater reach using: 1. Strengths-based messaging focusing on holistic gains, 2. Participant stories that highlight positive experiences, and 3. Peer support and information sharing to leverage altruism and curiosity. These theory-informed improvements will be used to increase participation in future trials, including people in hard-to-recruit groups. They may also inform other physical activity trials and community programs.

## Background

Regular physical activity (PA) has profound health benefits for older people, decreasing their risk of heart disease, diabetes, stroke, osteoporosis, depression, obesity, major cancers, falls and all-cause mortality, even when taken up in later life [1–5]. In addition to improved physical and mental function, participation in exercise programs can provide older people with structure, a sense of purpose, social connection and feelings of accomplishment [6, 7]. Over 60 motivators for older people’s engagement in PA have been identified, including physical and psychological benefits, pleasure, social interaction and increased self-efficacy [8].

Physical activity intervention trials play an essential role in informing public policy and health services design and delivery [9], yet trials promoting PA among older people frequently report low and skewed recruitment [10, 11]. Despite the pressing need to support older people with comorbidities and polypharmacy to exercise, PA trials frequently exclude or fail to recruit them [12], and many studies report lower rates of recruitment for older people from ethnic minorities, rural areas and other underserved communities [13–16]. Unrepresentative participation in research can reduce validity and generalisability [17, 18], and may contribute to inequalities in PA levels in hard-to-recruit groups [16].

Older people’s motivation to participate in PA trials has multiple dimensions and may be influenced by characteristics including sex, education level, self-efficacy and motivation readiness, existing health issues and levels of physical activity, relative age, socioeconomic status and cultural identification [8, 10, 15, 19–24]. Desire to address health goals, increase PA motivation and act altruistically are frequently reported incentives [25–27]. Reasons for older people declining to take part in PA trials include travel demands, lack of time, the belief that they are already sufficiently active, lack of interest in the research aims, unwillingness to be randomised and discomfort talking to researchers [16, 28].

The factors that influence older people’s decisions to participate in PA trials may differ from those of younger people. Frailty, comorbidities, fears about injury, and onerousness that outweighs benefits may dissuade them [29, 30]. Tai et al. note that there are few differences between younger and older people in terms of their beliefs about PA, but inactive older people have less intention to engage in PA, thus, *“Those most likely to benefit from exercise may be least likely to take it up”* [31:120]. This also applies to trial participation. Researchers conducting a randomised controlled trial (RCT) of PA advice for older people in general practice concluded that *“A continuing problem with recruiting participants for a project involving exercise is that the volunteer population tends to be healthy and interested in physical activity”* [32].

Well-designed exercise programs are effective in preventing falls in older people, and many PA intervention trials now incorporate fall prevention as a primary outcome [33–35]. Yet prevalent inaccurate beliefs about the causes and preventability of falls among older people deter many of those who could benefit [19, 36, 37]. Studies show that older people refuse participation in fall prevention interventions due to fear of falling, fatalism, denial, poor self-efficacy, stigma, overestimation of the effort required to participate, and both because they were too healthy and not healthy enough [19, 38, 39].

### What can trial recruiters do to encourage participation?

There are well-documented strategies for increasing participation in surveys [40] and clinical trials [41] but less empirical advice about how to successfully recruit older people for PA-based intervention trials [14]. Across all study types, high quality communication during recruitment is likely to be essential. Personalised contact has been found to encourage enrolment and continuance in various study types [26, 28, 38, 42, 43]. An RCT found that telephone contact from a researcher significantly increased recruitment of older people to a PA study [11], while participation in a multi-country fall prevention study was encouraged by a personal invitation from a health practitioner [44]. Conversely, while accessible study documentation is welcomed [26], invitation letters enhanced for readability and comprehension have not been found to affect recruitment rates [45]. Social media can be an effective platform for recruitment across all age groups [46].

Participation in interventions may also be incentivised by promoting attractive or beneficial intervention characteristics such as simplicity/usability, credibility, health monitoring, receiving motivational reminders, engaging interaction with the research team and, for those in trials, receiving progress reports and summary results of the research [27, 42, 43, 47, 48]. Where health professionals are used as recruiters there must be careful consideration of practice contexts and how the research is ‘sold’ to them. Studies across multiple RCTs have found that health professionals experienced organisational barriers to recruitment and many were sceptical about the likely intervention effectiveness and/or uncomfortable with aspects of the recruitment process [49, 50].

The role of trial recruitment strategies and their implementation is often overlooked; however, as Table 1 illustrates, studies are increasingly reporting on targeted strategies for reaching hard-to-recruit groups in PA trials.

**Table 1:** Some potentially effective strategies for reaching hard-to-recruit groups in physical activity trials.

Characteristics	Targeted recruitment strategies
Sedentary older people with health problems	Addressing physical symptoms and frailties in recruitment process, shorter interventions, tailoring interventions around existing health problems, promoting confidence in people’s PA abilities [10, 21]
The ‘oldest old’	Flexible interventions, gaining family support, emphasising the health benefits of PA, exploring people’s fears and preferences during recruitment [8, 15]
Older Indigenous people	Indigenous or culturally competent recruiters, culturally appropriate discussion (e.g. incorporate ‘yarning’), holistic and empowering interventions co-designed with the indigenous community, flexible participation, leveraging kinship and community connections, help with transport [22, 23]*
Older people of colour	Recruitment by trusted professionals/community leaders, appointment of a dedicated community health worker, targeting personal interests, face-to-face interaction, culturally appropriate communications [15, 24]
Older people in deprived areas	Peer recruitment and encouragement, providing refreshments, face-to-face interaction, appointment of a dedicated community health worker [15, 24]

*These strategies are extrapolated from the broader literature on indigenous people’s views of healthy ageing programs.

It is acknowledged that theory can usefully guide PA intervention design and can increase effectiveness, but most PA interventions for older people are not explicitly informed by theory and, where theories are cited, they are frequently used inconsistently or are not operationalised [51–53]. None, to our knowledge, have developed theoretical constructs that help to explain older people’s reasons for participating in PA trials and thus guide strategies for improved recruitment.

### Study aims

The purpose of this study was to explore why participants chose to enrol in the Coaching for Healthy Ageing (CHAnGE) trial [33], including how this decision may have been influenced by their experience of the recruitment process and if their expectations of the intervention were met. Better understanding of older participants’ reasons for enrolling in PA trials can help us anticipate barriers to participation and optimise the features of PA interventions that most appeal to older people, thereby improving intervention design and implementation practices, including recruitment and retention strategies [19, 27]. Qualitative methods are essential for developing rich understanding of people’s views and experiences [54] and the use of theory can provide a conceptual framework to guide this work [55] and increase the analytic generalisability of the findings [56]. We aimed to use self-determination theory as our framework. Self-determination theory (SDT) is well suited for investigating reasons for participation in interventions as it provides a conceptual structure for exploring anticipated needs satisfaction [57]. It has been used effectively to investigate older people’s motivation to participate in PA [58–61] and fall prevention programs [37].

### The Coaching for Healthy Ageing (CHAnGE) trial

The CHAnGE trial was a cluster randomised controlled trial testing the effectiveness of a healthy ageing program targeting inactivity and falls compared with a program targeting healthy eating. Participants were 72 groups of community-dwelling people aged 60 years and over based in a metropolitan centre and two regional towns in Australia. Groups were recruited from retirement villages and existing community organisations including Rotary clubs, Men’s Sheds, Probus and golf and bowling clubs. Presentations were given at community meetings by the research physiotherapists who would provide health coaching during the trial. Presentation content included information about the Australian PA guidelines, the benefits of PA in healthy ageing and fall prevention, and an overview of the CHAnGE trial. The presentation was designed to be highly accessible and was delivered informally with interaction and humour. Presenters facilitated a question and answer session afterwards and offered attendees the opportunity to enrol at that point or contact them later. Potential participants were provided with an information sheet that had been approved by the human research ethics committee overseeing the study. Precise records were not kept in all sites, but the recruiters estimate that between 20–45% of attendees enrolled in the trial. Importantly, those who enrolled appeared to be at the younger and more active end of the spectrum of potential trial participants at these meetings.

Recruited groups were randomised to either a healthy eating intervention involving written information and six months of fortnightly telephone health coaching from a government run “Get Healthy” service (the control arm) or to a PA and fall prevention intervention involving a home visit for fall risk assessment and advice, provision of an activity tracker, and fortnightly telephone health coaching by a research physiotherapist with training in health coaching (the intervention targeting the primary outcomes). Both interventions were delivered over 12 months and provided free of charge to participants. Primary outcomes were objectively-measured physical activity at 12 months post-randomisation and self-reported falls during the 12-month follow-up. Further details are available in the study protocol [33]. Trial registration details: ACTRN12614000016639, 07/01/2014.

## Methods

### Theoretical framework: Self-determination theory

Self-determination theory is increasingly used to inform intervention research in PA [62]. This theory proposes that human behaviour is driven by three interrelated needs which act as *“nutrients that are essential for well-being”* [57:213]: autonomy, competence and relatedness. People seek opportunities to meet these needs, and avoid threats to them, thus an intervention aimed at changing behaviour, and the new behaviour it seeks to embed, must satisfy these needs if it is to engage people and generate motivation [57, 63, 64]. Intervention strategies can satisfy these needs in different ways, but they do so by supporting existing capacities such as awareness, reflection, choice, reasoning and social interactivity [57]. See S1 Appendix for more detail.

Self-determination theory has been operationalised in the form of intervention techniques [65–67], and there is growing evidence of their efficacy [66]. A recent meta-analysis found that SDT-based interventions produced small-to-medium changes in most SDT constructs and in health behaviours at the end of the intervention period and at follow-up [68]. This theory can also guide the design and implementation of trial recruitment strategies, helping to highlight ways in which the intervention is likely to meet autonomy, competence and relatedness needs, and to avoid threatening them.

We chose to use SDT in this study because it aligns with self-regulatory behaviour change techniques featured in the CHAnGE intervention such as goal-setting and self-monitoring, and person-centred health coaching using motivational interviewing techniques, all of which are associated with effectiveness in PA interventions [69–73]. Autonomy, competence and relatedness are all threatened by unhealthy ageing, making SDT particularly appropriate for exploring how older people engage with PA:

*As our physical and mental capacities decline we are at risk of losing confidence and becoming more fearful*. *As we come to rely more on others for transport*, *shopping and other daily tasks*, *we can easily lose our feelings of control over our lives*. *With reducing capacities*, *we can also become more isolated and lonely in our homes and less able to make a significant contribution to others or society in general*. *[74*, *p*.*26]*

The current study utilises data from a wider qualitative process evaluation of the CHAnGE trial which was informed by SDT and which confirmed that the theory’s three core constructs were crucial to the intervention’s functioning [75].

### Participants and recruitment

The sample frame for this study comprised 127 CHAnGE trial participants from the PA and fall prevention arm who indicated on their 6-month follow-up evaluation form that they would be willing to participate in a semi-structured interview. Sampling was purposive to achieve maximum variation in interviewees’ age, socioeconomic status, geographic location and engagement with the intervention as perceived by their coach. The study physiotherapists/coaches reconfirmed willingness to participate with each individual and gave their contact details to the interviewers. Thirty-three people were invited but one declined due to health problems. Recruitment ceased when we met three thresholds: (i) our maximum variation sampling requirements were satisfied, (ii) early analysis indicated that the depth and quality of the data was sufficient to answer our research questions, and (iii) current interviews were not yielding important new conceptual information [76, 77].

### Data collection

Interview questions pertinent to this substudy focused on reasons for participation in CHAnGE. Interviewees were routinely asked, *“Why did you choose to take part in this program*?*”*. Where they gave responses that focused on extrinsic factors (e.g. accompanying a friend or supporting research) a follow-up question was asked, *“Were you hoping to get anything out of it for yourself*?*”*. Prompts that were informed by SDT were used to generate reflective in-depth responses about their reasons, including their perceptions of the recruitment process and any expectations of the intervention. More information, including the full interview guide, is available elsewhere [75].

Interviews were conducted by telephone by two experienced qualitative researchers, one male and one female, and were audio recorded. The interviewers had no prior relationships with the participants or the health coaches. To reduce social desirability bias, the participants were informed that the interviewers were not involved in the intervention design or delivery and that the health coaches would not have access to identifiable data. Audio recordings were professionally transcribed verbatim and checked for errors by the interviewers. Interviews took between 23 to 61 minutes with a mean duration of 37 minutes.

### Data analysis

Transcripts were analysed in NVivo 12 [78] using an interpretive description approach [79]. This approach incorporates common qualitative analytical techniques including immersion in the data, ‘constant comparison’ of data to identify thematic patterns and relationships across cases, and theorising about explanatory factors. The coding frame is available elsewhere [75]. A second round of thematic analysis was conducted for this substudy in which we inductively examined previously coded categories relating to recruitment and participation to identify themes regarding interviewees’ reasons for enrolment in CHAnGE [79, 80]. Lastly, a coding framework comprising the three main constructs from SDT (autonomy, relatedness and competence) was applied to these data to explore if and how the theory fit with and helped to explain our data. Throughout the evaluation study, research rigour was strengthened via independent coding, researcher triangulation, and critical review of developing themes including searching for disconfirming data as part of the constant comparative process [79, 81, 82].

### Ethics approval and consent

Ethical approval for this study was included in the CHAnGE trial approvals provided by The University of Sydney Human Research Ethics Committee, reference no. 2015/517. All trial participants gave prospective written informed consent at the trial commencement and verbal consent at the start of their interview. This included consent for their deidentified data to be used in publications.

## Results

A total of 32 participants were interviewed: 22 women (69%) and 10 men (31%), reflecting the 70/30 female/male ratio in the trial. Ages ranged from 60 to 82 years at the time of recruitment to the trial (mean 72, SD 6.2). Twenty-two participants lived in metropolitan Sydney and 10 in a regional centre. As determined by their home addresses, seven lived in suburbs with below average socioeconomic advantage, nine lived in suburbs with high socioeconomic advantage and the remaining 16 lived in mid-range suburbs [83]. Average daily steps at baseline ranged from 2,900 to 13,872 (mean 5,991) with only three interviewees taking more than 10,000 steps daily. Seven interviewees had fallen prior to the trial, two of whom each reported two falls. All interviewees were at least halfway (six months) through the intervention when interviews took place.

Value expectancy about CHAnGE at the time of enrolment focused on the program’s achievability, structured support, promotion of accountability and, in many cases, social connection. These were underpinned by interviewees’ belief that the intervention was likely to be effective because of the credibility of its design and of the physiotherapist health coaches who delivered it. Together these attributes were viewed as potential facilitators for program adherence and thus had the potential to improve health and fitness, including decelerating determinantal aspects of the ageing process such as minimising falls. The SDT concepts of competence, autonomy and relatedness were reflected strongly in this data which is now described in more detail. Illustrative quotes are used throughout this section. Details include interviewees’ sex and age at trial commencement, but quotes are otherwise de-identified to protect anonymity.

### Achievability

Interviewees felt that the CHAnGE recruitment process was clear and engaging, *“It was short*. *It was easy to understand*. *They didn’t go on and on*. *It was about things that are important to us and everyone was keen to listen and … it really sparked interest*.*”(female*, *71)*. Hearing that the intervention was designed for older people was a draw card for most interviewees who explained that the prospect of increasing PA with tired, older bodies was daunting—“*The ageing process has hit me*!*” (female*, *74)*. They welcomed the message that health coaches would have realistic expectations of participants and interpret activity data through a gerontological lens. This reassured interviewees that CHAnGE was achievable and would offer a safe entrée to PA, thereby supporting competence needs. The program’s flexibility (support for autonomy) seemed especially important for counteracting concerns that participation would be unmanageable: *“Some things you hear about*, *they’ve sounded too complicated or too hard whereas this one*, *I felt*, *sounded like it was something that I could do…*. *I could pull back if I needed to* …. *I could work it to suit my health plus my other interests” (female*, *76)*. Feelings of achievability were boosted for one 76-year-old male when he saw other, less able-bodied peers enrol: *“I was thinking if they can do it*, *probably I can do it”*. This also promoted a feeling of security, *“… if you’re in a group it’s easier*. *If you’re all doing something together it makes you feel safer”*.

Conversely, a few participants speculated that many older or less able-bodied members of their community groups who chose not to enrol were deterred by the physical challenge and, possibly, by confusion or worries about becoming entangled in onerous data collection:

*“… when I went to the first presentation on this at [my retirement village]*, *I got the impression that it sounded a bit daunting for the older people—people who are*, *say*, *ten years older than me*…. *Just looking around at the people and listening to their reactions* … *some of them were overwhelmed by this gigantic program” (male*, *70)*

### Structured support

The promise of structured support appealed to many interviewees as a way to boost competence and create accountability that would provide direction and motivation to work hard and stay on track:

*“I was lacking in confidence and I felt that having somebody take me through a program where I actually have to conform would be really good for me*. *It would make me stick to it* …. *just being structured I think*, *was the most important thing for me” (female*, *73)*.

Several associated their desire for structure with a life transition such as retirement, new working arrangements or recovery from illness. The timing of the trial was serendipitous for those who were looking for ways to establish new day-to-day routines that would also improve their health:

*“… leaving work is a big thing*. *You need something like this to happen otherwise* … *you might get very insulated*. *[With CHAnGE] I thought*, *you’ve got to get up and go for that walk because you’d think “Oh my goodness*, *I’ve got to get my steps up today”*. *It gets you out*. *Gets you about*. *Gets you talking to people*. *You can’t help it*, *you have to get out there*.*” (female*, *74)*

Some felt they had been paying insufficient attention to their health but were at a pivotal point where, with structured support, they could reorient towards self-care and self-prioritisation: *“It was about putting myself into my diary daily”* (male, 71).

The strategy of using the health coaches to present to social clubs was much appreciated and may have been crucial for conveying the message that CHAnGE would be supportive as well as structured. During these sessions health coaches modelled a friendly, empathetic manner which backed their claims that they would be encouraging (autonomy-supporting) rather than directive. It also helped meet relatedness needs by forging initial connections *“… she was the one at the [social club] who gave us the talk…*. *so you can put a face to the voice*, *you know*? *We sort of chatted and built up that rapport both ways*. *So it was good*.*” (male*, *77)*. Importantly, *t*hese recruitment sessions established CHAnGE’s credibility. Interviewees explained they could tell the intervention was evidence-based (i.e. it had *“some solid basis to it”*) and that the health coaches would be skilled in offering guidance about *“realistic activities and realistic goals” (female*, *65)* (competence supporting). This confidence was boosted by the intervention being offered as *“a research project through the uni” (female*, *71)*. This research context triggered curiosity in a few retired interviewees who identified this as a motivation to enrol in the trial: “*I was basically just interested in what it would be about*…. *I didn’t know whether it would be just exercise or nutrition or mental health or emotional health or what exactly the whole thing would entail” (female*, *75)*.

### Accountability

Interviewees talked about different forms of anticipated accountability. They expected regular calls from a health coach to create external accountability (the need to answer to someone), *“If you know someone’s looking over your shoulder and checking your results you try harder” (male*, *72)*. A 79 year-old woman for whom PA had previously been *“outside my interest range”* saw it as a chance to identify a realistic goal and then get both *“nagged”* and *“encouraged”* into working towards it. But many also regarded commitment to the trial as a form of accountability. Over a quarter of interviewees wanted to contribute to the study as a way of giving back to community, *“We were happy to take part in some research that was going to do somebody some good somewhere” (male*, *72)*. Most identified personal fitness benefits too, but a minority of those who described altruistic motivations said that supporting research was their only incentive. Several interviewees explained that contributing data to research made them keener to be a highly active participant. However, others expressed this as internal accountability, a sense of not letting oneself down, *“When you enter into a program you’ve made a commitment*…. *Even if it wasn’t a university study or anything like that it would be just the fact that if I make a commitment I follow it through” (male*, *64)*.

Interviewees emphasised structure and accountability far more in their reasons for enrolment than in their descriptions of what it was like to participate in the intervention, where flexibility (greater support for autonomy) and caring encouragement (an aspect of relatedness) seemed equally or more important for continuing engagement and motivation. This resonates with the spectrum of behavioural regulation proposed by SDT where competence-supporting autonomy and relatedness enable people to internalise the ‘rules’ of a practice and thus become more intrinsically motivated [63]. Indeed, findings from the wider evaluation of CHAnGE [75, 84] highlight interviewees’ sense of *“freedom”* in developing their own program and tackling it at times and in locations of their choosing:

*“*… *with this program I can choose whether to do a walk in the morning or last thing at night*, *so there’s that huge flexibility*…. *And the balance exercises can be done at home when you’re brushing your teeth or cutting up veggies*. *It’s good to be able to get the work in without having to commit to a place to go to do it*.*” (female*, *63)*

### Social connection

Interviewees described their supportive relationships with coaches as the central motivating feature of the intervention, highlighting the importance of relatedness in CHAnGE (we explore this in more depth in another publication [84]). However, this was not identified as an incentive at the point of enrolment where social connectivity with peers was regarded as the more important influence. The promise of social connection positively influenced enrolment in CHAnGE in three ways:

- Advancing existing social connections. Interviewees were supporting a friend or wanted to join others in their community group who had signed up: *“The fact that the people in the group were doing it as well made it more interesting for me*, *just because I’m a social person” (female*, *65)*.
- Enhanced accountability. Many interviewees hoped that obligation to other members of their recruitment group, combined with the program structure, would keep them motivated: *“[I was hoping for] some realistic activities to do … maybe in conjunction with other people too*. *I’m not really a self-starter*…. *left to my own devices there’s every chance it would just fall by the wayside” (female*, *67)*. Some were keen to find a PA ‘buddy’*—“Exercise is a lot more pleasurable when you’re with somebody else” (female*, *60)*.
- Enhanced social connections. The chance to meet people or develop closer relationships was a goal in itself for a few interviewees but this was less pronounced, possibly because they were aware that the intervention did not facilitate social gatherings.

### Improved health and fitness

The value of achievability, structured support and accountability was goal-orientated for most interviewees—focused on a desire to improve their general health and fitness. The multifaceted benefits of PA were recognised, including by those who reported that they struggled to stay active: *“*… *people just need physical activity to keep fit*, *to keep their minds active*…. *It makes you fitter and usually happier” (female*, *82)*. However, it was unclear to what extent the recruitment presentations conveyed this message. Many were already engaged in physical activity prior to the intervention and although most interviewees initially had a very open agenda about what their program might look like, several had already set personal goals which they saw the potential to incorporate and refine as part of the CHANGE intervention.

### Decelerated ageing

The SDT concepts of autonomy, competence and relatedness are evident in these desires for better physical functioning, especially in the emphasis interviewees placed on counteracting age-related physical, psychological and cognitive decline:

*“*… *one of the things that motivated me was the fact that at that particular time I was feeling low on energy*. *I was starting to feel really old* … *and I started to realise this is what could happen to me if I just let myself go*. *And I thought maybe this program could help*.*” (male*, *76)*

Many enjoyed activities that they felt were threatened by physical deterioration, including walking (and being in nature), exercise classes, golf, home repairs, social events, taking active holidays and playing with and caring for grandchildren. Independence, quality of life and dignity were primary concerns: *“I don’t want to be incapacitated for the wrong reasons*. *I’m proud and fiercely independent*, *I don’t want to have someone wiping my arse*.*” (male*, *71)*

A quarter of interviewees identified fall prevention as a key motivation for enrolment in CHAnGE. Five who had fallen before the trial were keen to learn strategies that could prevent recurrences: *“*… *the sense of powerlessness that I experienced when I had that first fall that ended up in an occult fracture and then the subsequent surgery—that’s gonna stay with me forever*. *I didn’t want it to happen again” (female*, *61)*. The recruitment presentation information on fall prevention resonated with these interviewees, *“It emphasised the necessity to be sure that I am strong enough and capable of keeping my balance” (female*, *74)*. However, two others who had fallen did not believe this required special attention: *“I didn’t feel the falls were a part of me—they were sort of accidents” (female*, *76)*. Others had noticed themselves becoming less stable and were alarmed at seeing or hearing about the effect of falls on peers. Those who lived in retirement villages seemed especially aware of the serious impact of falls.,

*“I didn’t have that problem but I had friends that had fallen and I’d seen the consequences*. *I realised as you get older you’re more likely to fall* … *As I said when I signed up to [CHAnGE]*, *I wanted to be as mobile as possible for as long as possible*.*” (female*, *76)*

### Did randomisation thwart autonomy?

The lack of choice inherent in randomisation did not appear to be a major barrier for any of the interviewees, although one woman expressed disappointment that she did not end up in the healthy eating arm of the trial. Only 10 people in the wider participant group withdrew immediately after being advised which trial arm they had been randomised to—eight of whom did so for health reasons—suggesting that this process did not compromise their sense of autonomy. However, lack of perceived autonomy in enrolment may have had a negative effect for the two interviewees who had been *“volunteered”* by their spouse and were purposively sampled for interviews because they were identified as having relatively low engagement in the intervention.

### Did CHAnGE meet interviewees’ expectations?

The majority of interviewees said that their largely positive expectations of the intervention had been met, especially by the combination of activity tracking and long-term, high quality health coaching. The CHAnGE outcome data are not yet available, but most of the 32 interviewees who took part in the qualitative process evaluation of this trial reported that the intervention had increased their PA levels, helped them to embed activities and generated positivity about PA [75]. In general, the intervention supported autonomy and competence needs, while relatedness was addressed in part by satisfying relationships with health coaches. But there were disappointments. The majority of these concerned the lack of PA-related peer support within their recruitment group. The CHAnGE investigators had hoped existing social connections within intervention recruitment groups might promote a shared interest in PA and a supportive local culture. However, while pre-existing PA-related social connections continued to flourish and to support CHAnGE activities, there were few examples of new connections being generated that would encourage greater PA.

## DISCUSSION

### How does self-determination theory help us understand people’s reasons for participating in CHAnGE?

Interviewees who sought to improve their fitness for health and fall prevention hoped that the intervention would offer structure and accountability to guide and motivate them. Self-determination theory asserts that structure acts as a supporting ‘scaffold’ for competence and self-regulation which, in turn, supports autonomy and increases motivation [57]. Positive structure is experienced as facilitative rather than controlling when it is based on clear and attainable objectives and is accompanied by empathetic and encouraging communication that reinforces competence (e.g. via positive feedback and advice) while promoting autonomy (e.g. emphasising choices for optimising goal attainment) [57, 63, 64, 73]. These characteristics align with the tenets of health coaching using motivational interviewing which was a central intervention component in CHAnGE [73, 85].

The change in participants’ descriptive emphasis from structure towards flexibility and relatedness as the intervention progressed suggests that interviewees developed increasing confidence in their abilities which satisfied competence needs. Further, that they were moving on a spectrum of behavioural regulation towards ‘identified regulation’ in which behaviours are autonomous and underpinned by consciously valuing physical activity as important to one’s life goals [64, 86].

Altruistic reasons for participating in trials have been noted in many other studies [e.g. 26, 27, 87]. Self-determination theory indicates that altruism and the sense of wellbeing it engenders is a synthesis of autonomy, competence and relatedness satisfactions, and highly favourable for intrinsic motivation [57]. The opportunity to influence future programs can be especially rewarding [88]. However, in most cases espoused altruism is accompanied by some level of self-interest. This has been described as *“conditional altruism”* [25] or *“weak altruism”* [89] whereby people are willing to help others providing they see some personal benefit and no major disadvantage. We also found that curiosity motivated some interviewees to enrol in CHAnGE. Curiosity is a natural feature of self-determination [57] and has also been identified as an incentive in other studies [20, 27, 48]. Tolmie et al. suggest that if curiosity is satisfied with pre-enrolment information it is likely to be superseded by other motivators such as altruism and personal benefit [47].

The CHAnGE intervention’s potential to enhance relatedness—group ‘belonging’ and/or PA-supportive social interaction—was an attraction for many interviewees. Although new peer group connections were not generated in most cases, our wider evaluation found that CHAnGE did capitalise on some existing connections, particularly where these had already formed around or included PA [75]. These relationships encouraged goal-attainment and sometimes provided practical support, and featured strongly in accounts of PA enjoyment. This concurs with other studies which suggest that socially supportive contexts can encourage and resource participation in both PA and fall prevention interventions [38, 43], and that relatedness needs do not necessarily have to be satisfied by health coaches or others in PA leadership roles if peers are actively engaged in supporting one another [57]. For example, exercise classes that emphasise peer cooperation can generate intrinsic motivation and greater enjoyment of PA [90]; while social encouragement is identified as a key facilitator for participating in fall prevention programs [38, 44]. Lee et al. report success in using social meetings as a strategy to encourage relatedness among older people in a group exercise program [91].

Unsurprisingly, interviewees were motivated to enrol in CHAnGE due to their expectation that it would benefit them. The concept of value expectancy is well established in other theories including the health belief model which has been used to inform physical activity intervention design and promotion [92–94]. It posits that motivation depends on assessment of the utility value and relative cost of expected outcomes. This encompasses competence/self-efficacy, the belief that ‘I can do this’, but adds judgments about ‘What will happen if I do?’ and, crucially, ‘Do I want to?’ [94]. Comments about achievability suggest that interviewees had *a priori* confidence that the work associated with CHAnGE would be within their capabilities, thereby satisfying competence needs, and that outcomes were likely to be positive due to the intervention’s credibility. But the question of desirability (‘Do I want to?’) may not have been optimally addressed. We explore the implications below, but note that it raises important questions about how to build perceived value and experiential value into intervention design and promotion. Thus the conceptual framework we would use to guide future work would use SDT and add the concept of value expectancy.

### Implications for intervention design and promotion

The community-based recruitment process clearly informed interviewees’ views about the CHAnGE trial and what they might get out of it. Our analysis suggests areas of strength but also areas for improvement.

#### Strengths-based messaging with greater emphasis on potential holistic gains from participation

The recruitment presentation explicitly promoted CHAnGE in terms of improved health and decelerated ageing, including fall prevention. It was unclear to what extent this was new information, but many interviewees suggested it aligned with existing views. They described a keen awareness of the threats of unhealthy ageing (albeit with very different views about their susceptibility to falling) and welcomed the intervention as a means of combating them. Although many saw fall prevention as a positive aspect of this, it did not tend to be a primary driver for enrolment. This suggests that fall prevention programs might be more attractive (and empowering) when they are framed holistically as healthy ageing programs. The potential for CHAnGE to contribute to healthier ageing was improved by perceptions of the intervention as structured, credible, achievable and, for some, as having the potential to leverage or boost social relationships. However, the relatively low uptake at most recruitment sessions suggests poor value expectancy for many, possibly affected by reservations about trial data collection and commitment. The tendency for enrolments across the trial to be at the younger and more active end of the spectrum of potential participants suggests that many older, frailer and relatively sedentary attendees at those presentations were not convinced that CHAnGE was right for them. Others report similar problems in recruitment for PA interventions and trials [11, 31, 32].

The tenets of SDT indicate that effective recruitment communication for PA and fall prevention interventions and trials should be strengths-based: emphasising capabilities and potential rather than deficits or threats to healthy ageing which are likely to undermine competence and autonomy. This aligns with the empowerment focus of CHAnGE and is supported by studies that indicate positive messaging is more motivating to older people [36, 58, 95]. For example, participants in a health promotion evaluation explained they switched off to health information if it gave them a *“bad conscience”* [88]. Consequently, strategies to encourage exercise in older people should target enabling factors including the benefits, affordability and safety of PA [53]. Hughes et al. [36] suggest messages that take account of past history and current limitations such as *“It is never too late to start being active”* and *“Be the best that you can be”*. While, in relation to fall prevention, Yardley et al. argue that,

*… older people do not reject falls prevention advice because of ignorance of their risk of falling, but because they see it as a potential threat to their identity and autonomy. Messages that focus on the positive benefits of improving balance may be more acceptable and effective than advice on falls prevention. [96, p*.*508]*

Attention to the concept of meaningful activity, rather than structure *per se*, is likely to resonate with many older people, especially those like our interviewees who were transitioning to retirement or recovering from illness, loss or depression and welcomed the intervention as a potentially positive focus in their lives. Routinised PA creates purpose, certainty and feelings of control and fulfilment for older people which may be especially rewarding in the aftermath of life-changing events [16, 97]. While SDT acknowledges that people have different motivations to engage in PA, some level of enjoyment is vital for sustained engagement [57, 94]. Enjoyment is often overlooked in PA intervention design, promotion and evaluation [97] yet our interviewees and many PA and fall prevention studies stress its importance [7, 35, 37, 53, 65, 90]. Consequently, recruitment for PA interventions and trials should enhance value expectations of PA by explicitly *“emphasizing engagement in meaningful activities*, *fostering self-determination*, *and enhancing sense of belonging and ‘mattering’ by supporting people to connect to their personal and collective strengths”* [98, p.369].

#### Use of participant stories to highlight positive experiences of the intervention

Data from the wider evaluation of CHAnGE indicates that the intervention successfully generated high quality relationships between participants and their health coaches, and that positive feedback from coaches during the intervention, combined with the personalised feedback from activity trackers, reinforced participants’ feelings of achievement and strengthened their motivation [75]. This indicates that autonomy, competence and relatedness needs were met. However, at the enrolment stage it was hard to convey how this would work—and, more specifically, how it would *feel*—particularly if participants failed to achieve their daily or weekly goals.

A key strength of the recruitment process was the onsite presentation by the health coaches which enabled potential participants to assess coaches’ personalities and interactive styles. It is likely that some threats to autonomy, competence and relatedness were minimised by coaches delivering information in an accessible manner, by emphasising terms such as ‘going at your own pace’, by demonstrating their gerontology expertise and realistic expectations of ageing bodies, and by their friendly interactions. However, while the presentation outlined the structure of CHAnGE it did not explicate the empowerment model of coaching used nor highlight its strengths in supporting self-determination. Therefore it is unclear to what extent participants could have assessed that CHAnGE would meet these fundamental needs.

The most powerful way to convey this information is likely to be participants’ own stories. Health service consumers’ stories are now widely used to provide rich insights for patient education, professional development, service redesign and intervention/trial content and evaluation [99–102]. Stories are relatable, increase perceived relevance and can change attitudes, behaviours and self-efficacy by modelling change processes and outcomes, and breaking down cognitive resistance so audiences are more open to information [102–104]. Other PA interventions use participant stories to motivate engagement with PA in general [105], but qualitative participant feedback data from pilots and previous similar interventions could also be used illustratively in trial recruitment as a persuasive tool, as well as informing wider trial implementation [106]. Previous participants could attend recruitment meetings to tell their stories and model ‘real world’ positive outcomes.

#### Leveraging altruism and curiosity with the promise of peer support opportunities and information sharing

A quarter of all interviewees said they participated in the intervention wholly or partly to contribute to the research, and honouring this commitment may have retained some who would otherwise have dropped out. Altruistic ‘community pay back’ motivation is well established in the trial literature [20, 25–27, 47, 89], including in relation to older people and exercise [7], but the question of whether pay back incentives could be harnessed through some other mechanism in scaled health interventions warrants more consideration. For example, many PA programs employ peer support by older people (e.g. [107, 108]), but little is known about the perceptions of and impacts on peer volunteers themselves in such programs. People are likely to experience autonomy, competence and relatedness satisfaction—and thus be more motivated—if their PA is viewed as beneficial both to themselves and others. So peer support roles may engender a sense of community pay-back that motivates the volunteers themselves to embed greater levels of activity. This indicates that interventions like CHAnGE could capitalise on altruism by incorporating strategies such as telephone-based peer support or exercise buddies.

Conditional altruism [25] suggests that recruitment promotes three areas: the potential value of the research, benefits to participants and how any disadvantages will be minimised. Benefits and disadvantages may differ for older people, many of whom will be retired and have fewer time demands but may find other features of the trial (e.g. travel or data collection) more onerous. Altruism, and the curiosity described by some of our interviewees, might be boosted by emphasising the novelty, purpose and longer-term goals of the research in recruitment materials, explaining that it is funded by a disinterested body (e.g. not a pharmaceutical company) and offering to make summary findings available to participants [17, 26, 87]. Tolmie et al. [47] found that an informal lunchtime meeting of researchers and participants, and an annual newsletter, were highly valued by older participants in their drug trial. The social aspects of the meeting were emphasised, but the communicative feedback loop provided by both these strategies is likely to have strengthened participants’ interest and sense of worth in contributing to the research.

Evidence from meta-analyses suggests that multiple co-acting intervention techniques used together to target all three SDT needs may be most effective in generating needs support [66] and building value-expectancy. This is likely to apply to recruitment also, including the use of messages that target different motivational incentives [58]. Consequently, autonomy, competence and relatedness should all be addressed, ideally via a suite of strategies. Some understanding of SDT will be required to operationalise the theory effectively [65].

### Limitations

Interviews were conducted at least 6 months into the intervention. This enabled us to explore the similarities and differences between interviewees’ expectations of the CHAnGE intervention and their actual experience of it, but interviewees’ memories of the recruitment process and their response to it may have faded in that time. The recruitment sessions were not observed by an independent researcher. This would have added another layer of rigour to our understanding of how they were delivered and received. Our interview data may be skewed towards positivity about CHAnGE given that it came from people who volunteered to take part in an interview. We did not interview people who declined to participate in the trial, or dropped out, and so do not have data about reasons for those choices including the role of self-determination in non-participation. Given that few members of the hard-to-recruit groups outlined in Table 1 participated in the trial, a considerable gap remains in our understanding of how to improve recruitment equity for CHAnGE.

## Conclusion

Recruitment strategies for the CHAnGE trial had many strengths, especially in using the intervention health coaches to deliver healthy ageing presentations in-person at community group meetings. However, it seems that more could have been done to promote the intervention in a way that addressed potential participants’ value expectancy and needs for self-determination. Health coaching and personalised activity tracking offered an empowering combination of self-directed (and, thus, achievable) program goals and activities, and respectful, person-centred support that provided encouragement and structured accountability. Once the intervention was underway, this combination appeared to satisfy most interviewees’ needs for autonomy and competence, and met relatedness needs insofar as they formed positive therapeutic alliances with health coaches. However, this potential was probably not conveyed optimally at the recruitment sessions or in the participant information, possibly leading to poorer understanding and uptake of the intervention, particularly by older/frailer people who may have felt daunted by the perceived demands of the intervention.

Drawing on SDT and its emphasis on satisfying three enduring needs—autonomy, competence and relatedness—we identify three strategies for improving the recruitment process for trials like CHAnGE: 1. Strengths-based messaging with greater emphasis on potential holistic gains from participation, 2. Use of participant stories to highlight positive experiences of the intervention, and 3. Leveraging altruism and curiosity with the promise of peer support opportunities and information sharing. Additional strategies may be required to boost equitable participation by people in hard-to-recruit groups. The approaches proposed in this study may also inform community-based PA programs and other types of trials.

## Supporting information

S1 Appendix A brief overview of self-determination theory and its implications for intervention design. (DOCX)

## Abbreviations

PA: Physical activity
RCT: Randomised controlled trial
SDT: Self-determination theory
