# Supplementary material for: Using self-determination theory to understand and improve recruitment for the Coaching for Healthy Ageing (CHAnGE) trial
Source: PLoS One. 2021 Nov 19;16(11):e0259873. doi: 10.1371/journal.pone.0259873 (PMC8604286; doi:10.1371/journal.pone.0259873)
Supplement: S1 Appendix — (DOCX) [file pone.0259873.s001.docx]

**S1 Appendix. A brief overview of self-determination theory and its implications for intervention design**

| **SDT construct** | **Some implications for intervention design** |
| --- | --- |
| **Autonomy:**  a full sense of volition and choice | Goal-setting and activity choice within the intervention must be self-directed and meaningful to each person. Participants should feel they ‘own’ their program and not feel pressured to adhere to a regime. External rewards or punishments are undermining and should be avoided. |
| **Competence:**  belief in one’s ability to use skills and attain desired outcomes | Intervention information should be accessible, actionable and useful to each person. Deliverers (such as health coaches) should demonstrate respect for participants’ intelligence, physical capabilities and ideas, and provide positive feedback on their progress. |
| **Relatedness:**  feeling close to, trusting of and cared for by others | Deliverers can build positive relationships via person-centred approaches characterised by empathy plus the meaningful choices and affirming feedback described above. Facilitating social connections that support PA can harness people’s tendency to internalise the values of their social group. Interventions do not have to provide positive relationships if participants have supportive social contexts. |
